# Diaqua­bis­{5-(pyridin-2-yl-κ*N*)-3-[4-(pyridin-4-yl)phen­yl]-1*H*-1,2,4-triazol-1-ido-κ*N* ^1^}iron(II)

**DOI:** 10.1107/S1600536813004601

**Published:** 2013-02-23

**Authors:** Bin Li

**Affiliations:** a Advanced Material Institute of Research, Department of Chemistry and Chemical Engineering, Qilu Normal University, Shandong 250013, People’s Republic of China

## Abstract

In the centrosymmetric title complex, [Fe(C_18_H_12_N_5_)_2_(H_2_O)_2_], the Fe^II^ ion, lying on an inversion centre, is coordinated by two *N*,*N*′-bidentate 5-(pyridin-2-yl)-3-[4-(pyridin-4-yl)phen­yl]-1*H*-1,2,4-triazol-1-ide ligands and two water mol­ecules in a *trans*-FeO_2_N_4_ geometry. In the ligand, the triazole ring makes dihedral angles of 5.21 (18) and 6.7 (2)°, respectively, with the adjacent pyridine and benzene rings. In the crystal, mol­ecules are linked by O—H⋯N hydrogen bonds, generating a three-dimensional network.

## Related literature

For background to coordination complexes, see: Zhang, Sun *et al.* (2012 ▶); Zhang, Fan *et al.* (2012 ▶); Fan *et al.* (2013 ▶).
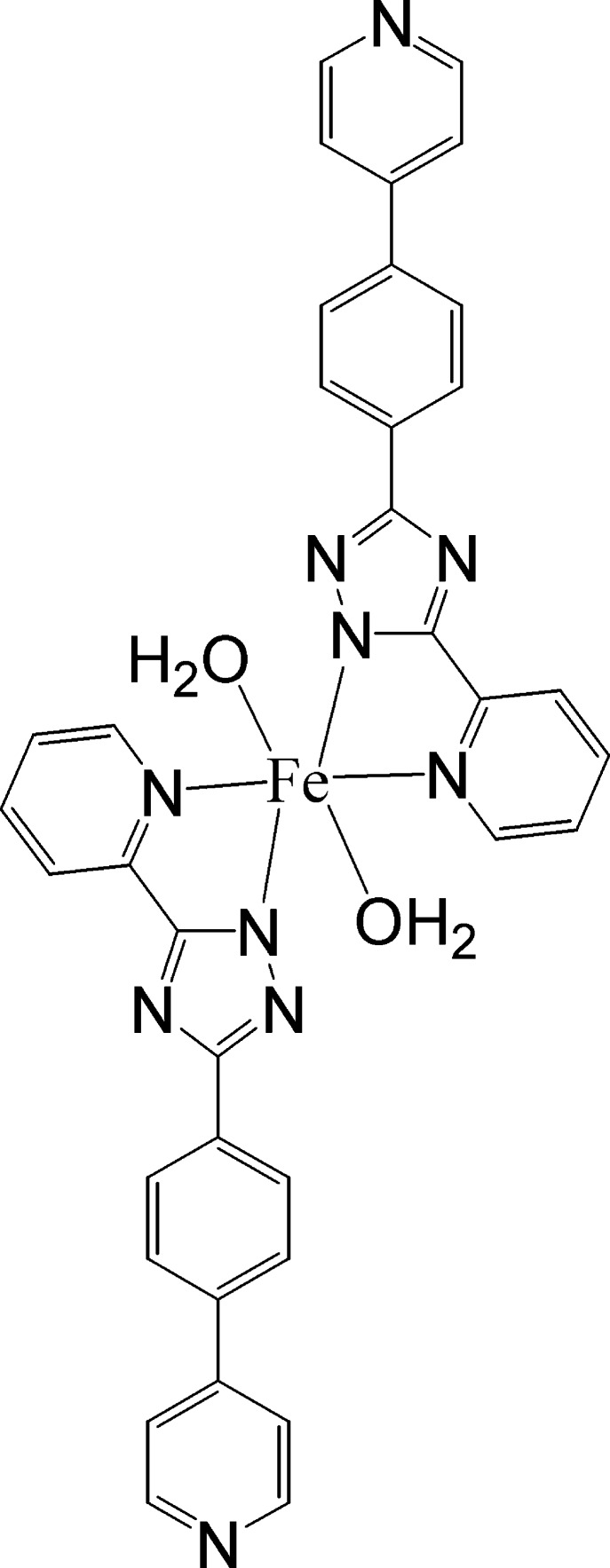


## Experimental

### 

#### Crystal data

[Fe(C_18_H_12_N_5_)_2_(H_2_O)_2_]
*M* *_r_* = 688.53
Monoclinic, 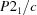

*a* = 13.1965 (17) Å
*b* = 12.0279 (16) Å
*c* = 9.9006 (13) Å
β = 100.998 (1)°
*V* = 1542.6 (4) Å^3^
*Z* = 2
Mo *K*α radiation
μ = 0.54 mm^−1^
*T* = 296 K
0.12 × 0.10 × 0.08 mm

#### Data collection

Bruker APEXII CCD diffractometer
Absorption correction: multi-scan (*SADABS*; Bruker, 2001 ▶) *T*_min_ = 0.938, *T*_max_ = 0.958
7711 measured reflections
2722 independent reflections
2214 reflections with *I* > 2σ(*I*)
*R*_int_ = 0.035

#### Refinement

*R*[*F* ^2^ > 2σ(*F* ^2^)] = 0.059
*wR*(*F* ^2^) = 0.184
*S* = 1.00
2722 reflections
231 parameters
1 restraint
H atoms treated by a mixture of independent and constrained refinement
Δρ_max_ = 1.14 e Å^−3^
Δρ_min_ = −0.42 e Å^−3^

### 

Data collection: *APEX2* (Bruker, 2004 ▶); cell refinement: *SAINT-Plus* (Bruker, 2001 ▶); data reduction: *SAINT-Plus*; program(s) used to solve structure: *SHELXS97* (Sheldrick, 2008 ▶); program(s) used to refine structure: *SHELXL97* (Sheldrick, 2008 ▶); molecular graphics: *SHELXTL* (Sheldrick, 2008 ▶); software used to prepare material for publication: *SHELXTL*.

## Supplementary Material

Crystal structure: contains datablock(s) global, I. DOI: 10.1107/S1600536813004601/is5245sup1.cif

Structure factors: contains datablock(s) I. DOI: 10.1107/S1600536813004601/is5245Isup2.hkl

Additional supplementary materials:  crystallographic information; 3D view; checkCIF report

## Figures and Tables

**Table 1:** Hydrogen-bond geometry (Å, °)

*D*—H⋯*A*	*D*—H	H⋯*A*	*D*⋯*A*	*D*—H⋯*A*
O1—H1*W*⋯N4^i^	0.81 (4)	1.99 (4)	2.784 (4)	168 (4)
O1—H2*W*⋯N5^ii^	0.85 (5)	2.39 (5)	3.165 (6)	152 (5)

Symmetry codes: (i) 
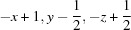
; (ii) 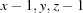.

**Fig. 1.**
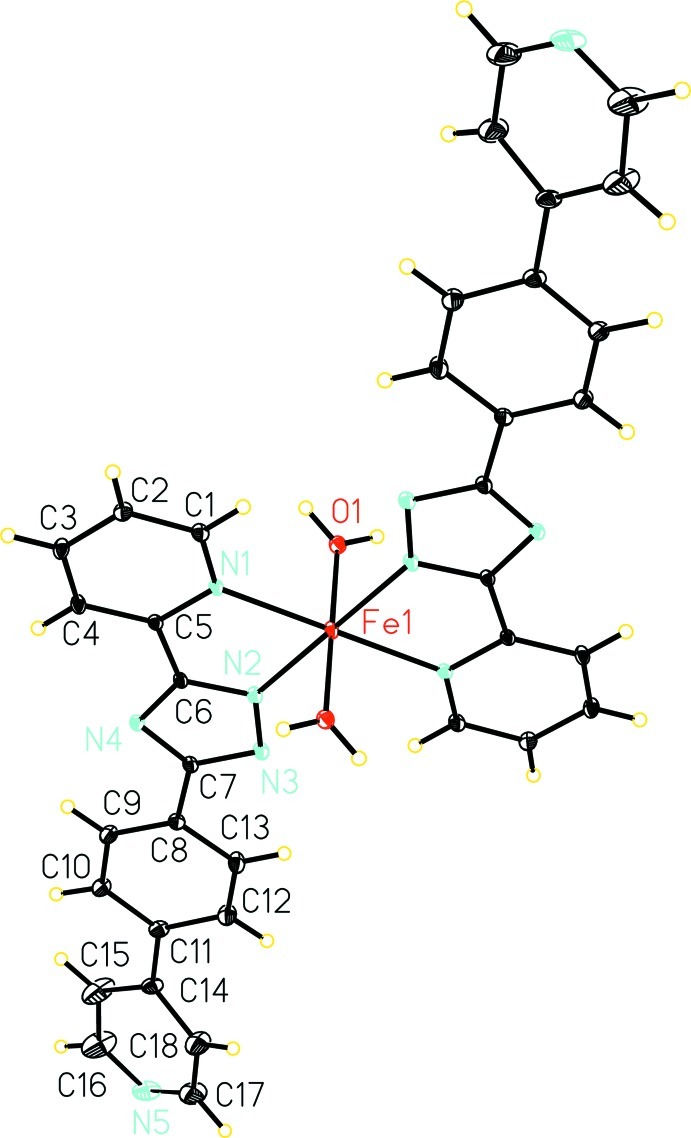
The molecular structure of the title compound with the atom-labelling scheme. Displacement ellipsoids are drawn at the 30% probability level. H atoms are given as spheres of arbitrary radius.

**Fig. 2.**
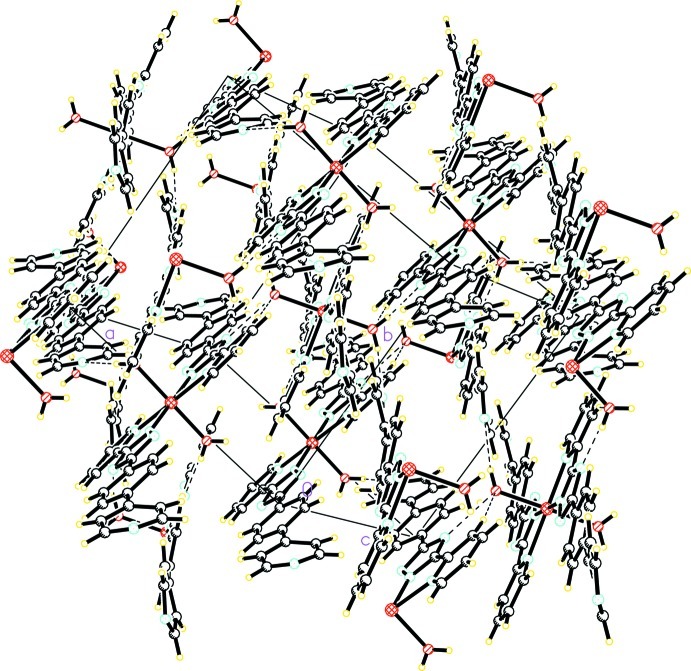
A crystal packing view of the title compound, displayed with hydrogen bonds as dashed lines.

## supplementary crystallographic information

### Comment

The design and synthesis of coordination complexes have attracted upsurging research interest not only because of their appealing structural and topological novelty but also owing to their tremendous potential applications in gas storage, microelectronics, ion exchange, chemical separations, nonlinear optics and heterogeneous catalysis (Zhang, Sun *et al.*, 2012; Zhang, Fan *et al.*, 2012; Fan *et al.*, 2013). Here, we report one new compound, [Fe(H_2_O)_2_(C_18_H_12_N_5_)_2_], obtained from the solvothermal reaction of 2-{3-[4-(pyridin-4-yl)phenyl]-1*H*-1,2,4-triazol-5-yl}pyridine and iron(II) chloride tetrahydrate.

The asymmetric unit of the title compound consists of a half of Fe(II), one deprotonated 2-{3-[4-(pyridin-4-yl)phenyl]-1*H*-1,2,4-triazol-5-}pyridine, and one water molecule. The Fe atom owns a distorted octahedral coordination geometry, completed by four N atoms from two deprotonated 2-{3-[4-(pyridin-4-yl)phenyl]-1*H*-1,2,4-triazol-5-yl}pyridine and two O atoms from two water molecules (Fig. 1). The Fe—O distance is 2.205 (3) Å. The Fe—N distances are 2.103 (3) and 2.171 (3) Å. The O—H···N hydrogen bonds (Table 1) in the crystal lead to a consolidation of the structure (Fig. 2) .

### Experimental

A mixture of 2-{3-[4-(pyridin-4-yl)phenyl]-1*H*-1,2,4-triazol-5-yl}pyridine (0.20 mmol, 0.060 g), iron(II) chloride tetrahydrate (0.40 mmol, 0.078 g), NaOH (0.20 mmol, 0.008 g) and 12 mL H_2_O was placed in a Teflon-lined stainless steel vessel, heated to 170 °C for 3 days, followed by slow cooling (a descent rate of 10 °C/h) to room temperature. Red crystals suitable for the X-ray experiment were obtained. Anal. Calc. for C_36_H_28_FeN_10_O_2_: C 62.80, H 4.10, N 20.34%; Found: C 62.72, H 4.03, N 20.18%.

### Refinement

All H atoms bound to C were refined using a riding model with C—H = 0.93 Å, and with *U*_iso_ = 1.2*U*_eq_ (C). The H atoms of the water molecule were located in a difference Fourier map and were refined with distance restraints of O—H = 0.820 (1) and H···H = 1.380 (1) Å [O—H = 0.81 (4) and 0.85 (5) Å].

### Crystal data

**Table:**

[Fe(C_18_H_12_N_5_)_2_(H_2_O)_2_]	*F*(000) = 712
*M**_r_* = 688.53	*D*_x_ = 1.482 Mg m^−^^3^
Monoclinic, *P*2_1_/*c*	Mo *K*α radiation, λ = 0.71073 Å
Hall symbol: -P 2ybc	Cell parameters from 425 reflections
*a* = 13.1965 (17) Å	θ = 2.3–16.0°
*b* = 12.0279 (16) Å	µ = 0.54 mm^−^^1^
*c* = 9.9006 (13) Å	*T* = 296 K
β = 100.998 (1)°	Block, colorless
*V* = 1542.6 (4) Å^3^	0.12 × 0.10 × 0.08 mm
*Z* = 2	

### Data collection

**Table:**

Bruker APEXII CCD diffractometer	2722 independent reflections
Radiation source: fine-focus sealed tube	2214 reflections with *I* > 2σ(*I*)
Graphite monochromator	*R*_int_ = 0.035
φ and ω scans	θ_max_ = 25.0°, θ_min_ = 2.3°
Absorption correction: multi-scan (*SADABS*; Bruker, 2001)	*h* = −11→15
*T*_min_ = 0.938, *T*_max_ = 0.958	*k* = −13→14
7711 measured reflections	*l* = −11→11

### Refinement

**Table:**

Refinement on *F*^2^	Primary atom site location: structure-invariant direct methods
Least-squares matrix: full	Secondary atom site location: difference Fourier map
*R*[*F*^2^ > 2σ(*F*^2^)] = 0.059	Hydrogen site location: inferred from neighbouring sites
*wR*(*F*^2^) = 0.184	H atoms treated by a mixture of independent and constrained refinement
*S* = 1.00	*w* = 1/[σ^2^(*F*_o_^2^) + (0.120*P*)^2^ + 1.3369*P*] where *P* = (*F*_o_^2^ + 2*F*_c_^2^)/3
2722 reflections	(Δ/σ)_max_ = 0.007
231 parameters	Δρ_max_ = 1.14 e Å^−^^3^
1 restraint	Δρ_min_ = −0.42 e Å^−^^3^

### Special details

**Table:**

Geometry. All esds (except the esd in the dihedral angle between two l.s. planes) are estimated using the full covariance matrix. The cell esds are taken into account individually in the estimation of esds in distances, angles and torsion angles; correlations between esds in cell parameters are only used when they are defined by crystal symmetry. An approximate (isotropic) treatment of cell esds is used for estimating esds involving l.s. planes.
Refinement. Refinement of F^2^ against ALL reflections. The weighted R-factor wR and goodness of fit S are based on F^2^, conventional R-factors R are based on F, with F set to zero for negative F^2^. The threshold expression of F^2^ > 2sigma(F^2^) is used only for calculating R-factors(gt) etc. and is not relevant to the choice of reflections for refinement. R-factors based on F^2^ are statistically about twice as large as those based on F, and R- factors based on ALL data will be even larger.

### Fractional atomic coordinates and isotropic or equivalent isotropic displacement parameters (Å^2^)

**Table:**

	*x*	*y*	*z*	*U*_iso_*/*U*_eq_	
C1	0.3747 (3)	0.2112 (3)	−0.1244 (4)	0.0363 (8)	
H1	0.3428	0.1627	−0.1925	0.044*	
C2	0.3486 (3)	0.3216 (3)	−0.1351 (4)	0.0412 (9)	
H2	0.3004	0.3476	−0.2095	0.049*	
C3	0.3951 (3)	0.3931 (3)	−0.0334 (4)	0.0425 (9)	
H3	0.3786	0.4683	−0.0383	0.051*	
C4	0.4663 (3)	0.3527 (3)	0.0757 (4)	0.0377 (9)	
H4	0.4977	0.4000	0.1455	0.045*	
C5	0.4900 (2)	0.2410 (3)	0.0795 (3)	0.0285 (7)	
C6	0.5674 (2)	0.1872 (3)	0.1860 (3)	0.0280 (7)	
C7	0.6842 (3)	0.1449 (3)	0.3520 (3)	0.0310 (8)	
C8	0.7662 (3)	0.1451 (3)	0.4768 (3)	0.0357 (8)	
C9	0.7863 (3)	0.2334 (4)	0.5670 (4)	0.0468 (10)	
H9	0.7465	0.2976	0.5507	0.056*	
C10	0.8648 (3)	0.2276 (4)	0.6809 (4)	0.0512 (11)	
H10	0.8764	0.2881	0.7403	0.061*	
C11	0.9267 (3)	0.1348 (4)	0.7094 (4)	0.0456 (10)	
C12	0.9037 (4)	0.0443 (4)	0.6214 (5)	0.0576 (12)	
H12	0.9421	−0.0207	0.6395	0.069*	
C13	0.8253 (4)	0.0489 (3)	0.5086 (5)	0.0557 (12)	
H13	0.8112	−0.0132	0.4522	0.067*	
C14	1.0144 (3)	0.1269 (4)	0.8279 (4)	0.0515 (11)	
C15	1.0280 (5)	0.1932 (7)	0.9391 (6)	0.124 (3)	
H15	0.9809	0.2496	0.9455	0.149*	
C16	1.1133 (6)	0.1770 (8)	1.0451 (6)	0.132 (4)	
H16	1.1206	0.2259	1.1191	0.158*	
C17	1.1777 (5)	0.0489 (6)	0.9320 (7)	0.0927 (19)	
H17	1.2319	0.0018	0.9229	0.111*	
C18	1.0973 (5)	0.0595 (6)	0.8208 (6)	0.0885 (19)	
H18	1.0993	0.0205	0.7403	0.106*	
Fe1	0.5000	0.0000	0.0000	0.0319 (3)	
N1	0.4444 (2)	0.1702 (2)	−0.0198 (3)	0.0300 (6)	
N2	0.5878 (2)	0.0804 (2)	0.1719 (3)	0.0335 (7)	
N3	0.6647 (2)	0.0523 (2)	0.2789 (3)	0.0371 (7)	
N4	0.6261 (2)	0.2328 (2)	0.2991 (3)	0.0307 (7)	
N5	1.1827 (3)	0.1012 (5)	1.0503 (4)	0.0787 (14)	
O1	0.3849 (2)	−0.0453 (3)	0.1252 (3)	0.0406 (6)	
H1W	0.374 (3)	−0.110 (4)	0.140 (5)	0.063 (15)*	
H2W	0.336 (4)	−0.002 (3)	0.136 (5)	0.055 (15)*	

### Atomic displacement parameters (Å^2^)

**Table:**

	*U*^11^	*U*^22^	*U*^33^	*U*^12^	*U*^13^	*U*^23^
C1	0.040 (2)	0.0363 (18)	0.0265 (18)	−0.0005 (16)	−0.0088 (15)	−0.0023 (15)
C2	0.044 (2)	0.038 (2)	0.034 (2)	0.0095 (17)	−0.0115 (16)	0.0047 (16)
C3	0.054 (2)	0.0281 (18)	0.040 (2)	0.0084 (17)	−0.0064 (17)	0.0031 (15)
C4	0.047 (2)	0.0277 (17)	0.0325 (19)	−0.0005 (15)	−0.0077 (16)	−0.0042 (15)
C5	0.0304 (17)	0.0278 (16)	0.0244 (17)	−0.0019 (13)	−0.0024 (13)	−0.0018 (13)
C6	0.0319 (17)	0.0238 (16)	0.0248 (17)	−0.0027 (13)	−0.0036 (13)	0.0009 (13)
C7	0.0350 (18)	0.0304 (17)	0.0234 (17)	−0.0018 (14)	−0.0046 (14)	−0.0003 (14)
C8	0.0352 (19)	0.0381 (19)	0.0283 (18)	0.0013 (15)	−0.0077 (14)	−0.0014 (15)
C9	0.044 (2)	0.051 (2)	0.038 (2)	0.0121 (18)	−0.0089 (17)	−0.0124 (18)
C10	0.044 (2)	0.063 (3)	0.039 (2)	0.012 (2)	−0.0115 (17)	−0.022 (2)
C11	0.038 (2)	0.064 (3)	0.0303 (19)	0.0060 (19)	−0.0057 (16)	−0.0057 (19)
C12	0.058 (3)	0.049 (2)	0.054 (3)	0.013 (2)	−0.020 (2)	0.000 (2)
C13	0.062 (3)	0.040 (2)	0.052 (3)	0.007 (2)	−0.023 (2)	−0.009 (2)
C14	0.036 (2)	0.075 (3)	0.038 (2)	0.008 (2)	−0.0067 (17)	−0.005 (2)
C15	0.088 (4)	0.196 (8)	0.064 (4)	0.067 (5)	−0.048 (3)	−0.063 (5)
C16	0.094 (5)	0.225 (10)	0.056 (4)	0.048 (6)	−0.038 (3)	−0.061 (5)
C17	0.063 (4)	0.120 (5)	0.080 (4)	0.018 (4)	−0.024 (3)	0.002 (4)
C18	0.071 (4)	0.107 (5)	0.072 (4)	0.028 (3)	−0.025 (3)	−0.016 (3)
Fe1	0.0380 (5)	0.0226 (4)	0.0274 (4)	0.0003 (3)	−0.0131 (3)	−0.0020 (3)
N1	0.0311 (15)	0.0258 (14)	0.0277 (14)	−0.0008 (11)	−0.0080 (11)	−0.0002 (11)
N2	0.0377 (16)	0.0274 (14)	0.0278 (15)	0.0006 (12)	−0.0127 (12)	0.0005 (12)
N3	0.0397 (17)	0.0308 (15)	0.0317 (16)	0.0013 (13)	−0.0158 (13)	−0.0009 (13)
N4	0.0320 (15)	0.0299 (14)	0.0260 (14)	0.0000 (12)	−0.0054 (12)	−0.0047 (12)
N5	0.050 (2)	0.130 (4)	0.045 (2)	0.010 (3)	−0.0188 (18)	0.004 (3)
O1	0.0414 (16)	0.0322 (15)	0.0438 (15)	0.0010 (13)	−0.0031 (12)	0.0047 (13)

### Geometric parameters (Å, º)

**Table:**

C1—N1	1.341 (4)	C11—C12	1.391 (6)
C1—C2	1.371 (5)	C11—C14	1.485 (5)
C1—H1	0.9300	C12—C13	1.371 (6)
C2—C3	1.376 (5)	C12—H12	0.9300
C2—H2	0.9300	C13—H13	0.9300
C3—C4	1.378 (5)	C14—C15	1.344 (7)
C3—H3	0.9300	C14—C18	1.374 (7)
C4—C5	1.378 (5)	C15—C16	1.398 (7)
C4—H4	0.9300	C15—H15	0.9300
C5—N1	1.352 (4)	C16—N5	1.287 (9)
C5—C6	1.470 (4)	C16—H16	0.9300
C6—N2	1.325 (4)	C17—N5	1.320 (8)
C6—N4	1.350 (4)	C17—C18	1.381 (7)
C7—N3	1.327 (4)	C17—H17	0.9300
C7—N4	1.351 (4)	C18—H18	0.9300
C7—C8	1.479 (4)	Fe1—N2	2.103 (3)
C8—C9	1.380 (5)	Fe1—N1	2.171 (3)
C8—C13	1.397 (5)	Fe1—O1	2.205 (3)
C9—C10	1.380 (5)	N2—N3	1.363 (4)
C9—H9	0.9300	O1—H1W	0.81 (4)
C10—C11	1.381 (6)	O1—H2W	0.85 (5)
C10—H10	0.9300		
			
N1—C1—C2	122.8 (3)	C18—C14—C11	120.4 (4)
N1—C1—H1	118.6	C14—C15—C16	119.6 (6)
C2—C1—H1	118.6	C14—C15—H15	120.2
C1—C2—C3	118.5 (3)	C16—C15—H15	120.2
C1—C2—H2	120.7	N5—C16—C15	126.4 (6)
C3—C2—H2	120.7	N5—C16—H16	116.8
C2—C3—C4	119.7 (3)	C15—C16—H16	116.8
C2—C3—H3	120.1	N5—C17—C18	124.3 (6)
C4—C3—H3	120.1	N5—C17—H17	117.8
C5—C4—C3	118.9 (3)	C18—C17—H17	117.8
C5—C4—H4	120.6	C14—C18—C17	120.8 (6)
C3—C4—H4	120.6	C14—C18—H18	119.6
N1—C5—C4	121.8 (3)	C17—C18—H18	119.6
N1—C5—C6	113.3 (3)	N2—Fe1—N2^i^	180.0 (2)
C4—C5—C6	124.9 (3)	N2—Fe1—N1^i^	103.68 (10)
N2—C6—N4	112.6 (3)	N2^i^—Fe1—N1^i^	76.32 (10)
N2—C6—C5	118.6 (3)	N2—Fe1—N1	76.32 (10)
N4—C6—C5	128.8 (3)	N2^i^—Fe1—N1	103.68 (10)
N3—C7—N4	114.1 (3)	N1^i^—Fe1—N1	180.00 (14)
N3—C7—C8	119.4 (3)	N2—Fe1—O1^i^	90.52 (11)
N4—C7—C8	126.4 (3)	N2^i^—Fe1—O1^i^	89.48 (11)
C9—C8—C13	117.5 (3)	N1^i^—Fe1—O1^i^	91.52 (11)
C9—C8—C7	124.1 (3)	N1—Fe1—O1^i^	88.48 (11)
C13—C8—C7	118.4 (3)	N2—Fe1—O1	89.48 (11)
C10—C9—C8	120.7 (4)	N2^i^—Fe1—O1	90.52 (11)
C10—C9—H9	119.6	N1^i^—Fe1—O1	88.48 (11)
C8—C9—H9	119.6	N1—Fe1—O1	91.52 (11)
C9—C10—C11	122.1 (4)	O1^i^—Fe1—O1	180.00 (19)
C9—C10—H10	119.0	C1—N1—C5	118.3 (3)
C11—C10—H10	119.0	C1—N1—Fe1	125.9 (2)
C10—C11—C12	117.0 (3)	C5—N1—Fe1	115.6 (2)
C10—C11—C14	123.7 (4)	C6—N2—N3	107.2 (3)
C12—C11—C14	119.3 (4)	C6—N2—Fe1	116.1 (2)
C13—C12—C11	121.3 (4)	N3—N2—Fe1	136.7 (2)
C13—C12—H12	119.4	C7—N3—N2	104.7 (3)
C11—C12—H12	119.4	C6—N4—C7	101.4 (3)
C12—C13—C8	121.3 (4)	C16—N5—C17	112.8 (5)
C12—C13—H13	119.4	Fe1—O1—H1W	121 (3)
C8—C13—H13	119.4	Fe1—O1—H2W	123 (3)
C15—C14—C18	114.1 (4)	H1W—O1—H2W	113 (4)
C15—C14—C11	124.8 (4)		

Symmetry code: (i) −*x*+1, −*y*, −*z*.

### Hydrogen-bond geometry (Å, º)

**Table:**

*D*—H···*A*	*D*—H	H···*A*	*D*···*A*	*D*—H···*A*
O1—H1*W*···N4^ii^	0.81 (4)	1.99 (4)	2.784 (4)	168 (4)
O1—H2*W*···N5^iii^	0.85 (5)	2.39 (5)	3.165 (6)	152 (5)

Symmetry codes: (ii) −*x*+1, *y*−1/2, −*z*+1/2; (iii) *x*−1, *y*, *z*−1.
